# Monitoring mental distress in Para athletes in preparation, during and after the Beijing Paralympic Games 2022: A 22 week prospective mixed-method study

**DOI:** 10.3389/fspor.2022.945073

**Published:** 2022-10-11

**Authors:** Marte Bentzen, Göran Kenttä, Tommy Karls, Kristina Fagher

**Affiliations:** ^1^Department of Teacher Education and Outdoor Studies, The Norwegian School of Sport Sciences, Oslo, Norway; ^2^The Swedish School of Sport and Health Sciences, Stockholm, Sweden; ^3^School of Human Kinetics, University of Ottawa, Ottawa, ON, Canada; ^4^Swedish Paralympic Committee, Stockholm, Sweden; ^5^Rehabilitation Medicine Research Group, Department of Health Sciences, Lund University, Lund, Sweden

**Keywords:** depression, anxiety, Paralympic Games, mental disorder (disease), sport for persons with disability, Para athletes, monitoring athlete health

## Abstract

It is common in elite sport to monitor athletes' training load, injuries and illnesses, but mental distress is rarely included. An improved understanding of the epidemiology of mental distress among elite Para athletes and how their coaches perceive such monitoring would allow us to better develop and implement preventive measures. The purpose of this study was therefore to (1) prospectively describe elite Para athletes' mental distress, before, during and after the Beijing Paralympic Games (Paralympics Games 22 = PG22); and to (2) gain a better understanding of *if* and *potentially how* awareness of athletes' mental distress changed, through weekly monitoring, and influenced how coachers perceive athletes' mental distress and if they accounted for this before, during and after PG22. A mixed-method study design was used, in which prospective mental distress (depression and anxiety) data were collected weekly from 13 [Swedish] elite Para athletes in preparation, during and after PG22. Data were screened and evaluated weekly by a physiotherapist and a sports psychologist, and coaches also received weekly reports. A focus-group interview with the coaches were conducted post Paralympics to address coaches' awareness about mental distress and athlete health monitoring in Parasport. For data analyses, descriptive statistics was used for the quantitative data and a content analysis was conducted for the qualitative data. The results reveled the following proportion of datapoints indicating symptoms of anxiety and depression: before PG22 (15.8 and 19.1%); during PG22 (47.6 and 38.2%); and after PG22 (0 and 11.8%). The qualitative results indicated that coaches perceived athlete health monitoring as helpful for increasing their awareness of mental distress, and as a useful tool to initiate support for their athletes as well as improving their coaching. In summary, this cohort of elite Para athletes reported a high proportion of mental distress during the Winter Paralympic Games in Beijing. The results also show that it is important and feasible to monitor Para athletes' mental distress to detect and manage early symptoms of mental distress.

## Introduction

The Paralympic Games is one of the largest multi-sport events in the world, hosting the world leading athletes with impairments to compete (1, 2). The interest of para sport has increased tremendously the past decade, and the elite para sport context is nowadays a high-performance context including a wide range of organizational- and performance-oriented demands and stressors that may affect the athlete (3, 4). Some parts of the Paralympic cycle are said to be more demanding than others, such as qualifying for the Paralympic Games, and ultimately performing at one's best during the Paralympics (4–6). Still, the knowledge about athletes' mental health during the Paralympic Games is scarce even though increasing attention has been given to mental health in sport during the past decade (7).

A recent systematic review estimated that the prevalence of mental distress symptoms in able-bodied elite athletes is 19% for general psychological distress and/or alcohol misuse, 26.4% for sleep disturbance, and 34% for anxiety/depression (8). To mitigate the risk of mental distress that athletes may face in an elite sport context when trying to balance the challenges of high performance and demands it has been suggested to further assess mental distress in sports, and especially in Para sport (4, 9, 10).

In addition to the stressors that all elite athletes are exposed to, research has suggested that Para athletes are exposed to additional stressors such as discrimination (11), insufficient and non-adapted sport facilities, additional costs regarding impairment-specific equipment (12), logistical challenges related to travel to competition sites, “misclassification” for competitions (9), and poor access to specialized and adequate health care (13). Also, injuries and illnesses are found to be major mental stressors for all elite athletes, and mental health issues can increase risk of injuries (14). Despite significant efforts in developing injury and illness preventive measures, recent data suggest an increasing trend of sports injuries and illnesses in several populations along with compressed competitions schedules (15, 16). Monitoring injuries and illnesses is an established procedure in many elite sports settings (17). However, few athlete health monitoring systems have included mental health (18), despite it could be hypothesized that both injuries, illnesses, a high training load and a demanding life of being an elite athlete could be associated.

Another concern is that more injuries and illnesses have been reported during the recent Paralympic Games compared to the Olympic Games (16, 19). Additionally, Para athletes have a pre-existing impairment that may negatively affect both physical and mental health (18, 20).

Still, a recent study looking specifically at research about mental distress symptoms in Para sport found that only seven previous studies have targeted Para athletes (9). The findings showed that most studies are small scale using non-standardized measures of mental distress (9). Only three studies included defined measures of depression (21–23) and one anxiety (21, 22, 24), whereas the rest of the studies explored psychological constructs such as identity and self, stress and wellbeing (9).

Another stressor prior the 2022 Paralympic Games (PG22) was the challenges related to the ongoing COVID-19 pandemic, and the strict protocols implemented in China (25, 26). The COVID-19 pandemic has influenced the everyday life of elite athletes, and reported stressors include an overall insecurity related to changing plans, restrictions from traveling and competing, postponement and/or cancellation of major events, physical health issues related to the infection, and problems with support systems (27–29). During the COVID lockdowns, disruptions in everyday routines for able-bodied athletes as well as isolation have been associated with increased depression, anxiety and stress symptoms (30). It has also been suggested that especially Para athletes were more detrimentally affected by increased levels of perceived stress during the COVID lockdown period compared to Olympic athletes (31, 32). Overall, there is a paucity in the literature concerning the epidemiology of mental distress in elite Para athletes (33), and an improved understanding would allow us to better develop and implement preventive measures targeting Para athletes.

Another important aspect is coaches' awareness and actions regarding their athletes' mental health. Elite coaches play an important role in the lives of their athletes, both in terms of providing competence and guidance in relation to sport specific performance enhancement, but also in relation to giving emotional support over time (34, 35). The quality of the coach-athlete relationship, with a focus on the emotional support and overall wellbeing of the athlete has been shown to be of significance for improved performance in both Olympic (36) and Paralympic athletes (37). Nowadays, many coaches are aware of how to prepare Para athletes with mental training before larger events such as the Paralympic Games (38, 39). Still, it is in recent position papers about mental health in sports argued that coaches' awareness regarding mental health and safeguarding is lacking [e.g., (6, 40)]. An improved understanding about coaches' awareness of their athletes' mental distress and how they monitor and communicate about mental distress would allow us to better understand how to detect early symptoms of mental distress among elite Para athletes.

Taken together, the aim of this study was to (1) prospectively describe Para athletes' mental distress before, during and after PG22; and to (2) gain a better understanding of *if* and *potentially how* increased awareness of athletes' mental distress through weekly monitoring influenced how coachers perceive athletes' mental distress, and if they accounted for this during preparation and performance phases of PG22.

## Methods

### Study design

This study used a sequential mixed-method research design (41, 42) including a prospective longitudinal quantitative data collection of athletes' mental distress, followed by a qualitative data collection including coaches' experiences and awareness of their athletes' mental distress and their own coaching over time. As mental distress is a complex topic with many interacting factors, it can be hypothesized that the combination of systematic and empirical data with narrative and holistic data can improve our understanding of athlete distress in Parasport. The combination of quantitative and qualitative research is also supported by current trends within sports medicine [e.g., (43)], and in sports and health psychology [e.g., (44, 45)] when exploring complex and understudied research questions. For the current study, a sequential quantitative → qualitative approach was deliberately chosen. The arrow indicates that the quantitative data influences the qualitative data (46).

### Participants and recruitment

In September 2021, all [Swedish] elite winter Para athletes (*N* = 13) that were in the national training squad and in the [BLINDED] Paralympic program were invited to participate in this study. The following inclusion criteria were used: (a) being able to communicate in [Swedish]; (b) age 18–65 years; (c) having the ability to respond to a weekly online survey and (d) being an elite Para athlete that had the potential to qualify for the PG22. This was a convenience sample, and all athletes from this total population accepted to participate. Ten of the athletes qualified for the Paralympic Games, two were substitutes at home, and one athlete who were on track to qualify but did not make it in the last qualification competition very close up to the Games. In addition, one athlete who was supposed to travel to PG22 tested positive for COVID-19 right before departure and had to stay at home.

The head coaches for the included athletes were also invited to participate in the study. All three coaches accepted the invitation and agreed to participate in the qualitative focus group interview 2 weeks after returning from PG22.

### Ethical considerations

Ethical approval was obtained from the [Swedish] Ethical Review Authority (2021-05827-01). The project followed the World Medical Association's Declaration of Helsinki Ethical Principles (47). Participation in the study was voluntary, informed consent was obtained and the participants could at any time terminate their study participation without giving a reason. Data were collected and stored according to the General Data Protection Regulation. The project follows the Strengthening the Reporting of Observational Studies in Epidemiology (STROBE) guidelines (48), and the Consolidated Criteria for Reporting Qualitative Research (COREQ) (49).

### Quantitative data collection

The prospective self-report data from the athletes was collected weekly from November 2021, over 22 weeks; 16 weeks during the preparation of PG22, 3 weeks during precamp and PG22, and 3 weeks after returning from PG22. The data collection followed the recommendations in “The Para sports translation of the IOC consensus on recording and reporting of data of injury and illness in sport” (50). Data was collected in an adapted and accessible eHealth-based application developed, evaluated and used in the Sports-Related Injury and Illness in Paralympic Sport Study (SRIIPSS) (13, 20, 51).

Every Sunday, the athletes received a web survey *via* email and/or text message with questions regarding their previous training week. If a respondent did not answer the questionnaire, one reminder was sent. The following questions were included in the weekly report: hours of training, rate of exertion, quality of training, hours of sleep, mental distress, nutrition the past week, and any new injury or illness in the past week. The mental distress parameter used for the current study was the measure of mental health adapted from the Patient Health Questionnaire (PHQ-4). The PHQ-4 is a brief screening scale with four items in total for anxiety (e.g., “Feeling nervous, anxious or on the edge”) and depression (e.g., “Feeling down, depressed or hopeless”) (52). PHQ-4 has been used in other studies in sports medicine (30, 53, 54). The participants were asked to report how often they had been bothered with the following problems on this scale in the last week: 0 = Not at all; 1 = Several days; 2 = More than half the days; 3 = Nearly every day. A total score of the two items, respectively, tapping into anxiety and depression was calculated. A total score ≥ 3 for the scales is defined as anxiety and depression (52). This measure is considered a valid measure of detecting both anxiety and depression (52), and has been previously used in studies of athletes [e.g., (30, 55)]. It should be noted that the original version of the PHQ-4 scale is validated to capture the frequency of participants experiences the last 2 weeks (52). However, this study was designed to weekly monitor athletes' distress, which is established procedure in athlete health monitoring (50, 56). Also, another recent study in sports medicine have used PHQ-4 with weekly measurement points (53).

The quantitative data was screened and evaluated weekly primarily by a physiotherapist specialized in sports medicine and a sports psychologist. All athletes that reported a new injury, illness or two or more items on the PHQ-4 scale were contacted by phone by the physiotherapist or sports psychologist to follow up on their health status. Each week the national team head coach for each sport received a summary about their athletes' health.

### Qualitative data collection

A focus group interview was then used to collect qualitative data from the Paralympic head coaches. A focus group interview is described as “a carefully planned discussion designed to obtain perceptions on a defined area of interest in a permissive, non-threatening environment” [(57), p. 2], and is considered a suitable method to gain deeper insight into commonly held opinions within a group (58). Importantly, the head coaches were invited to engage from the start in the initiative of monitoring their athletes prior, during and after the PG22, and to this end were provided with weekly status reports on their athletes. Nevertheless, it was expected that they would have their own individual experiences related to the same protocol and event. As such, it was hypothesized that a focus group interview would facilitate a discussion among participants, both related to commonly shared experiences, but also enable discussion regarding different perceptions of the protocol and events. As the head coaches knew each other well, it was expected that they could openly share potential disagreements more easily, in contrast to focus group interviews where participants are not familiar with each other (58). Thus, the researchers played the role of moderators in the conversation during the interview, encouraging participants to contribute their experiences on the topics in the interview guide (59).

Prior to the focus group interview, researchers conducted a preliminary screening of the quantitative data. When conducting the interview, the researchers were familiar with the summarized individual reports, both prior to PG22 and during PG22. The second author conducted the interview, while authors three and four were present in the room. The interview guide was centered around three topics, with open questions for the participants to share their experiences and opinions. These topics were: experiences with the weekly screenings and how this potentially influenced their coaching; awareness regarding mental distress in Para sport; and lessons learned from participating in the protocol/initiative. The researcher asked probing and clarification questions where needed. The interviewer has an extensive research background in conducting interviews, in addition to training in counseling. Moreover, the researchers experience covered applied work in coach education and coach development, including experience from two Paralympic Games. This background and contextual knowledge were constructive and supportive during the interview for at least two reasons: to make the participants feel comfortable, and to probe matters of interest.

The focus group interview was held in a conference room that was set up for audio-recording in March 2022. The interview was audio taped and it lasted for 55 min.

### Data analyses

To describe the population, demographic data concerning gender, sport, type of impairment and age were used. Mental distress data were analyzed using descriptive statistics. Pearson's correlation (*r*) were used to control for the relationship between the two variables of mental distress due to possible comorbidity among anxiety and depression, and the strength of the correlation coefficient *r* were guided by the following cut-offs: trivial (*r* < 0.1), small (0.1 < *r* < 0.3), moderate (0.3 < *r* < 0.5), large (0.5 < *r* < 0.7), very large (0.7 < *r* < 0.9) and nearly perfect (*r* ≥ 0.9) (60).

The sample was relatively small (*N* = 13), yet every participant was asked to answer the questionnaire 22 times. As such, the strength in this design is not expressed by the number of participants alone, however, in relation to the number of participants x number of times of data collection (61). For the longitudinal quantitative data collection over 22 weeks, the response rate was in total 81.5%. Response rates for the three periods of data collection were: Pre PG22 (16 weeks: 86%); during PG22 (3 weeks: 53.8%); after PG22 (3 weeks: 87%). The number of datapoints per participant is displayed in Table 1. In accordance with guidelines for scoring the PHQ-4 (52), the total score of the subscales anxiety and depression was interpreted as: “≥ 3” = clear symptoms; “2” = mild symptoms; “0–1” = no symptoms. The results on how many weeks each participant reported these three categorizations of symptoms in each of the three periods of the study (pre, during and post PG22) were calculated and displayed to illustrate the variations in individual profiles. In addition, these results were summarized to give an overview of how many of the weeks in total in each of the three periods of the study (pre, during and post PG22) and calculate an overall percentage for the total population.

**Table 1:** Overview of weeks with symptoms of anxiety and depression before, during and after PG22.

	**Pre Paralympics (16 weeks)**							**During Paralympics (3 weeks)**							**After Paralympics (3 weeks)**						
**Level symp**		**0–1**		**2**		**≥3**			**0–1**		**2**		**≥3**			**0–1**		**2**		**≥3**	
	**N^dp^**	**Ax**	**Dp**	**Ax**	**Dp**	**Ax**	**Dp**	**N^dp^**	**Ax**	**Dp**	**Ax**	**Dp**	**Ax**	**Dp**	**N^dp^**	**Ax**	**Dp**	**Ax**	**Dp**	**Ax**	**Dp**
**ID**
1	16	16	16	0	0	0	0	0	0	0	0	0	0	0	3	3	3	0	0	0	0
2	16	9	7	3	6	4	3	3	0	1	0	2	3	0	3	3	3	0	0	0	0
3	15	14	14	1	1	0	0	3	3	3	0	0	0	0	3	3	3	0	0	0	0
4	14	14	14	0	0	0	0	0	0	0	0	0	0	0	2	2	2	0	0	0	0
5	16	13	16	3	0	0	0	2	2	2	0	0	0	0	3	3	2	0	1	0	0
6	13	13	13	0	0	0	0	3	3	3	0	0	0	0	3	3	3	0	0	0	0
7	16	16	16	0	0	0	0	1	1	1	0	0	0	0	3	3	3	0	0	0	0
8	16	9	0	2	5	5	11	3	0	0	0	0	3	3	3	3	0	0	1	0	2
9	16	16	16	0	0	0	0	3	2	3	1	0	0	0	3	3	3	0	0	0	0
10	4	3	3	1	1	0	0	0	0	0	0	0	0	0	1	1	1	0	0	0	0
11	6	5	5	1	1	0	0	0	0	0	0	0	0	0	1	1	1	0	0	0	0
12	16	13	15	3	1	0	0	0	0	0	0	0	0	0	3	3	0	0	1	0	2
13	14	9	9	3	3	2	2	3	0	0	1	2	2	1	3	3	2	0	1	0	0
N^dp^ in total	178	150	144	17	18	11	16	21	11	13	2	4	8	4	34	34	26	0	4	0	4
%		84.3	80.9	9.6	10.1	6.2	9.0		52.4	61.9	9.5	19.1	38.1	19.1		100	76.4	0	11.8	0	11.8

N^dp^: Number Data Points; Ax, Anxiety; Dp, Depression; Level symp, Level symptoms: 0–1 = No symptoms; 2 = Mild symptom; ≥3 = Clear symptoms.

Content analysis was used to analyze the qualitative data (62). The audio recorded interview was first transcribed and reread to become familiar with the content. The analyses could be described as abductive because it included both deductive and inductive stages (63). As such, the initial themes were created both deductively from the structure of the interview guide and inductively from meaning making of patterns from the raw data. The content analysis initially revealed five subthemes, however, during discussion among the researchers in charge of the data analyses, it was agreed that these subthemes had overlapping content. As such, the initial five subthemes were merged, and three overarching themes were created in the final stage of the analysis as presented in the results.

## Results

### Demographics

The sample of athletes consisted in total of 13 athletes, eight males and five females. Three participants were in the 18–25 years old age group, three participants were in the 26–35 years old age group, while seven participants were above 36 years old. Twelve participants had a physical impairment, while one participant had a visual impairment. The athletes were active in the following sports: para alpine skiing, para cross country skiing and wheelchair curling. The coach sample consisted of three male head-coaches, within the age range of 29–53 years. All of them had previous experience coaching in Paralympic Games, altogether a total of seven times. In terms of objective performance outcomes for this sample of participants for the PG22, in total, they won two gold medals, two silver medals and three bronze medals.

### Results from quantitative data

The descriptive results for all weeks of reported levels of symptoms of anxiety and depression before, during and after PG22 are displayed in Table 1. The correlation between anxiety and depression were positively and largely associated (*r* = 0.57, *p* < 0.001).

Over the 16 weeks before PG22, participants answered in total 178 times on measures of anxiety and depression. Of these datapoints, on average, the majority of participants reported no symptoms of anxiety (84.3%) and depression (80.9%). In total, 9.6% reported mild symptoms of anxiety, 10.1% mild symptoms of depression, while 6.2% of the datapoints showed participants scoring for clear symptoms (i.e., more severe) of anxiety, and 9% for depression. In total, it was three of the thirteen participants that reported clear symptoms of both anxiety and depression during the 16 weeks before PG22 (ID 2, 8, and 13). For one of the participants (ID 8), clear symptoms of both anxiety and depression were reported for several weeks in row. For the other two participants (ID2 and 8), the higher scorings were more evenly spread over the 16 weeks.

During the three-week period of PG22, participants answered in total 21 times on the measures of anxiety and depression. It should be noted that four of the participants for different reasons (COVID, substitutes, and did not qualify) did not travel to PG22, and their number of datapoints have therefore been set to 0 (non-responding) for these 3 weeks. It was decided to set their score to 0, and not to omit their data for these 3 weeks. The reason for this was because it was valuable to keep their data in this study for the 16 weeks prior PG22 and 3 weeks after PG22, and at the same time secure anonymity of not revealing the ID of the four not traveling to PG22. Of the valid datapoints, on average, 56.7% of the participants' datapoints reported no symptoms of anxiety, and 61.9% reported no symptoms of depression. Mild symptoms of anxiety were reported in 9.5% of the datapoints, and for mild symptoms of depression in 19.1% of the datapoints. Clear symptoms of anxiety were reported in 38.1% of the datapoints and clear symptoms of depression in 19.1% of the datapoints. Of the 13 participants, it was three of the participants that reported clear symptoms of anxiety (ID 2, 8, and 13) and of these three two of them that reported clear symptoms of depression during PG22 (ID 8 and 13). During PG22, with only 3 weeks of measurements, all three of these participants reported clear symptoms of anxiety and/or depression in either two or three consecutive weeks.

Over the 3 weeks after the PG22, the participants in total provided answers on 34 datapoints on the measures of anxiety and depression. Of these datapoints, 100% indicated no symptoms of anxiety and 76.4% indicated no symptoms of depression. Further, 11.8% of the datapoints indicated mild symptoms of depression and 11.8% indicated clear symptoms of depression. After PG22, none of the participants reported clear symptoms of anxiety, while two participants reported clear symptoms of depression (ID 8 and 12). After PG22, with only 3 weeks of measurements, the two participants with clear symptoms of depression reported this in two consecutive weeks.

### Results from qualitative data

**Figure 1:**
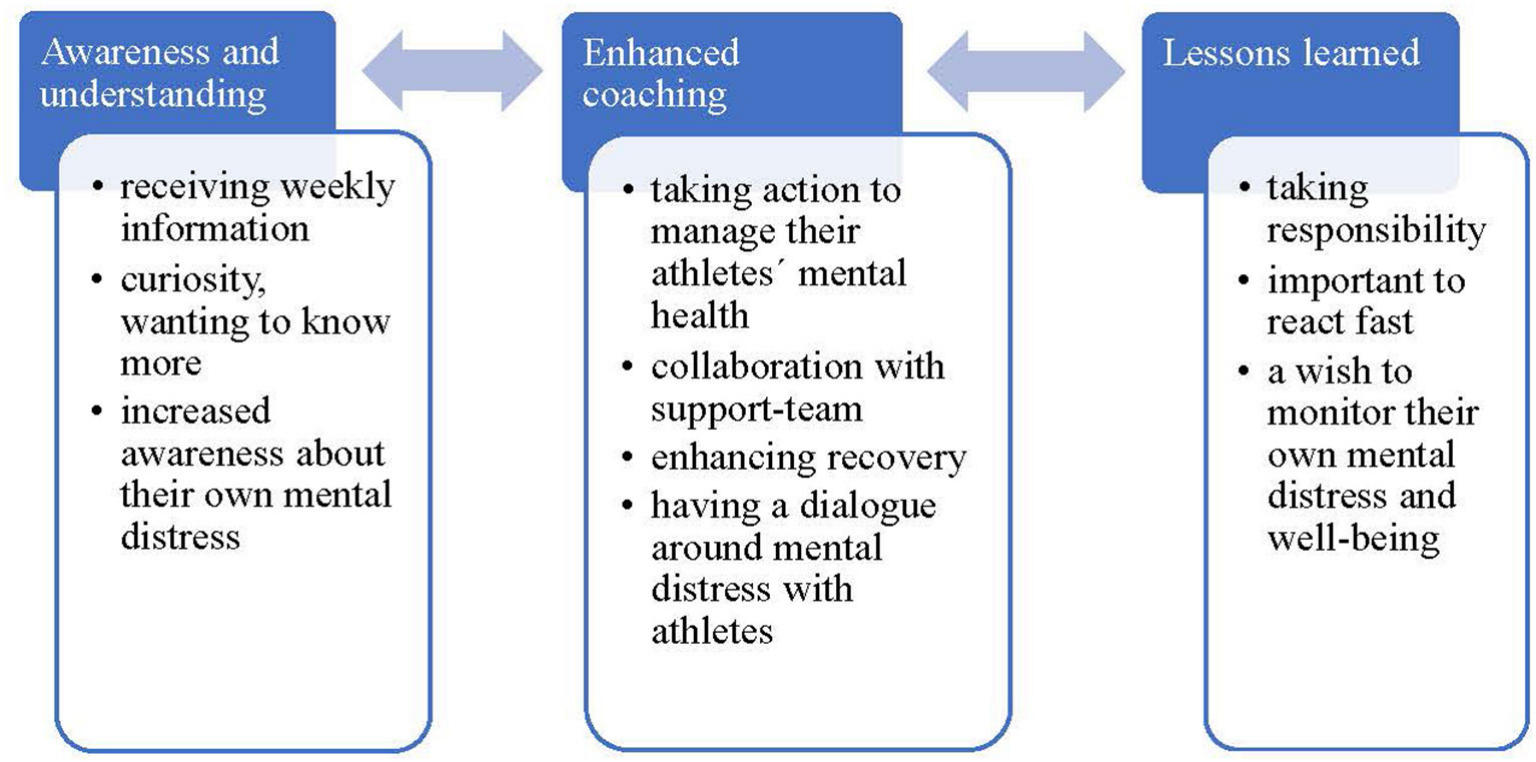
How coaches perceive monitoring of their athletes' mental distress.

The findings of the qualitative analyses concern how coaches perceive monitoring of their athletes' mental distress revealed the following three overarching themes; (1) awareness and understanding; (2) enhanced coaching; and (3) lessons learned. These themes are also presented in Figure 1 to provide a conceptual understanding.

### Awareness and understanding

Overall, this monitoring process organically developed an increased awareness and understanding of their athletes' self-rated mental distress. However, several examples illustrated contextual differences. For example, some coaches appreciated receiving the knowledge through the weekly reports that otherwise may have passed unnoticed. Other coaches gained deeper knowledge through close collaboration with a sport psychologist attached to their team. One coach noted: “*The ratings sometimes confirmed something that I kind of sensed was coming*”. Importantly, all coaches also developed a qualitatively different view regarding mental distress that was expressed by curiosity, wanting to know more, increased awareness about their own mental distress, reflecting upon, and how there still remains a need to lower the threshold for help-seeking. Altogether, as illustrated the arrow in Figure 1, the theme of awareness and understanding is interdependent with the next theme, enhanced coaching.

### Enhanced coaching

The theme of enhanced coaching became evident through several examples, such as taking action to manage their athletes' mental health based on when “red flags” (i.e., screened high on mental distress symptoms) were reported. This was typically done in close collaboration with sport psychology support, but also with members of the coaching staff. These actions occurred during the preparation leading up to the Paralympic Games. As an example, one coach said, “*Great to have a source of information, making it easier to know when and how to approach the athlete.”*

Another coach described taking action by enhancing recovery through minimizing training load. Other actions targeted team dynamics, and some focused on “keep it simple” and maintaining rehearsed routines. Noteworthy, the coach approached the athlete to address their mental health, either directly or after a first contact with the sport psychologist. The latter was specifically discussed in relation to various strategies, i.e., best practice, that did aim to validate self-report ratings of mental distress symptoms with personal communication. The coaches concluded that some athletes were more open in self-report ratings, and others in personal communication. The perception was that it became easier for the coaches to have a dialogue around mental distress especially with athletes that normally kept quiet about their mental distress when they reported their health status in the monitoring system. Moreover, all coaches provided examples of closer collaboration both with support staff, the coaching team, and with the athletes because of monitoring the athletes' mental distress. Nevertheless, one coach discussed a temporary challenge and the need to work extremely hard in order to maintain a collaborative team climate and performance at the expense of less attention to athlete health monitoring during a time when team selection becomes an issue prior to the Paralympic Games.

### Lessons learned

Finally, as suggested by the arrows in Figure 1 it can be argued that the theme “lessons learned” positively influence the theme enhanced coaching and vice versa. All coaches reflected upon how important and critical the start-up phase of athlete health monitoring is and how it requires clear and thorough information about the monitoring system. It is especially important to make it clear to everyone involved, who will have access to data and warrant that action will always be taken in the best interest of the athletes. Further, all coaches discussed the value of responding fast when the weekly report revealed either a red flag or a warning sign. However, time constraints may be a challenge as noted by one coach: “*It is fundamental to have enough time to engage and oversee the monitoring system to make the best use of it as a fish-net”*. A somewhat unexpected finding came out during the final reflection in the focus group in that all coaches talked about how they would see a value in monitoring their own mental distress and wellbeing.

## Discussion

The aim of the current study was to prospectively explore Para athletes' mental distress on a weekly basis before, during and after PG22, which potentially is their most stressful period in their performance cycle. A secondary aim was to better understand *if* and *potentially how* awareness of athletes' mental distress changed, through the use of athlete health monitoring, and influenced how coaches perceive athletes' mental distress and its relationship to preparation and performance during PG22.

To the best of our knowledge this is the first study that has prospectively monitored symptoms/signs of anxiety and depression among Para athletes in connection to the Paralympic Games. In addition, it includes data on how coaches perceived such monitoring.

### The overall findings of monitoring elite para athletes' mental distress

Athlete health monitoring is a fundamental element of concerted efforts to protect athletes' health (64). Still, most studies have focused on monitoring sports injuries in Para athletes, despite that it is recommended to also monitor “any state of health problems” in this population (50). It has been suggested that there are barriers and insecurities of studying mental distress in individuals with a disability (9). For example, the [Swedish] Public Health Agency only measures physical health among persons with a disability in their yearly national health survey, whereas both physical and mental health is assessed among individuals without a disability (65). Thus, an important finding from this study is that the results show that it is possible and feasible to monitor Para athletes' mental distress over time. The response rate was 85%, which is considered a good response rate in epidemiological studies (66). In addition, all the coaches' perceived it valuable for performance and wellbeing to monitor their Paralympic athletes' distress. Based on the results from this study it can, thus, be recommended to continuously monitor elite Para athletes' mental distress.

### Para athletes' mental distress before, during and after a paralympic games

During the preparation period *before* PG22, 9.6% of the athletes reported mild symptoms of anxiety, and 6.2% reported clear symptoms of anxiety. Overall, 10.1% reported mild symptoms and 9% reported clear symptoms of depression. Combined, these results are similar to Swedish able-bodied elite athletes, in which it has been shown that 19.5% reached the clinical cut-offs for symptoms of anxiety and/or depression (67). Notable, is that that this study used the full length of PHQ-9 and GAD-7 to measure anxiety/depression, but a strength is the comparison of two settings from the same country. Also, a recent study from Australia shows that elite Para athletes report the same mental health and wellbeing rates as elite able-bodied athletes (68). Taken, together these results suggest that Para athletes and able-bodied elite athletes report similar rates of mental distress.

Concerning the mental distress *during* PG22, the athletes reported mild symptoms of anxiety in 9.5% of the datapoints, and clear symptoms of anxiety in 38.1% of the datapoints. For depression, both mild and clear symptoms of depression were reported in 19.1% of the datapoints. Even though this study only includes a small sample, the results indicate that large competitions, such as the Paralympic Games, are stressful events [e.g., (4)]. Noteworthy, the results also indicate that the mental distress symptoms are more prevalent than physical illnesses during the Paralympic Games, for example, 13.6% reported a physiological illness during the 2018 winter Paralympic Games (16). Furthermore, the results demonstrate the importance of including mental health support from both the local organizing committee's medical support and the National Paralympic committee's medical team (4). Overall, there is a paucity of studies evaluating athletes' mental distress during large competitions such as the Paralympic and Olympic Games, and to the best of our knowledge this is the first study that has monitored para athletes' mental distress *during* a Paralympic Game. Based on these findings it is recommended that future studies include larger cohorts of athletes to evaluate Para athletes' mental distress, its association to performance, the specific sport and other health parameters *during* major competitions.

Taking a person-centered approach when studying the results, it became evident that it was three out of thirteen athletes who reported clear symptoms of anxiety and depression before and during the Paralympic Games. Two of these athletes had more infrequent reportings of clear symtoms througout the 16 weeks before the Paralympic Games. Based on clinical research it is important that early symptoms of mental distress is noticed by either coaches or members of the support team, which enable early interventions that is more effiecent than treatment at a later stage. Importantly, one of the participants reported clear and prolonged symptoms of mental distress, which indicates a case that needs follow up assessment and possibly referal to professional treatment (69). More strikingly from a narrow-minded performance perspective, the same three athletes also reported clear sympoms during the Paralymic Games. From an applied perspective it is a challenge that may involve ethically informed choices when there is a need to support both mental health and performance during major competitive events such as the Paralympic and Olympic Games.

Three weeks *after* PG22, the athletes in this population reported low levels of anxiety and depression. Consequently, it could be hypothezied that stress levels as well as mental and emotional pressure fade away when returning home. However, two participants reported clear symptoms of depression after PG22. Therefore, it is important that the medical and high performance team provide support to athletes prior and during the competition, but also post competition. In addition to this stance, previous studies among Olympic athletes indicate that returning from the Olympic Games can lead to so called “Post Olympic blues”, in which the athlete may experience a crisis transition leading to mental health issues (70). Factors that may influence the risk of developing “Post Olympic blues”, irrespective of the athlete's performance, have been linked to unreasonable expectations, media intrusion, internal conflicts, feelings of isolation and failure to meet their own and others' expectaitons (70). Another suggested explanation to the post-Olympics-blues, that goes beyond performance, is the major contrast between the perceived highs at the Games and the lows when returning home (71). Yet, there is no data on “Post Paralympic blues” and it is recommended to further assess this phenomenon also in Para sport.

### Mental distress during the COVID-19 pandemic

Another important aspect to highlight is that the results from the current study should be interpreted in light of the ongoing COVID-situation during the Beijing Paralympic Games 2022, and it was prior to the Games suggested that the strict isolation protocols during the Paralympic Games could expose some individuals to mental distress (25). However, the protocols may have managed spread of infection since only 26 individuals tested positive during the Paralympic Games (in total 150,815 tests were conducted on athletes and officials) (72). None of the athletes or officials from [Sweden] tested positive during the Games. Moreover, to evaluate the actual impact of the pandemic and the strict protocols on mental distress during the Paralympic Games the results of the current study need to be compared to future studies. Furthermore, it is important to address that [Sweden] did not have any lockdown during the pandemic, which allowed most elite athletes to continue to train as usual. A study conducted in Norway during the pandemic, a country with a similar COVID-strategy, found that Paralympic/Olympic athletes reported lower levels of depression and anxiety symptoms compared with semi-elite athletes (73). It was suggested that especially elite athletes were not affected that much due to a strong support team (73). However, this study did not do separate analysis on Olympic and Paralympic athletes. Also a recent study from Germany assessed elite Para athletes' mental health during the pandemic, and it was demonstrated that lower PHQ-4 values were reported by the para-athletes compared to the general population (53).

### Coaches' experiences of monitoring their athletes' mental distress

Awareness and understanding of mental distress have the potential to lower the threshold for help-seeking, thus facilitating recovery and return to competition. Based on the qualitative findings from this study it can be argued that health monitoring that targets athletes' mental distress, and provides weekly information to their coaches as well as, integrated support together can act as a vehicle to organically build awareness and understanding of mental distress among coaches. Moreover, this resonates with previous research that has emphasized the importance of developing mental health literacy (7). Future research should therefore also examine the athletes' perceptions and experiences of monitoring their own mental distress with attention to how it relates to develop context specific mental health literacy.

The coach-athlete relationship is perhaps the most intense relationship in sport, in which a coach's and an athlete's cognitions, feelings and behaviors are mutually and causally interrelated (74, 75). Interestingly, findings in the current study suggest that all coaches reported enhanced coaching based on taking actions and developing a closer collaboration with not only athletes, but also the coach-team and the support-teams based on monitoring mental distress. Importantly, this finding may counter-balance the research on coach mental health reporting that several coach stressors are associated with managing their athletes' (76). Based on the results from this study it can therefore be hypothesized that monitoring athlete health has the potential of improving the coach-athlete relationship and reducing coaches' stressors. In addition, these various examples of improved coach-performance are likely to also enhance athlete performance.

However, it is crucial to also carefully address two challenges that implicitly were discussed between the coaches, that is confidentiality and team-selections. Athletes need to feel totally comfortable that their self-ratings will only be used with the best of interest to sustain their health without interfering with team-selections. Therefore, providing clear information and consent that rule out any risk of misuse is essential to sustain a monitoring system of mental distress in elite sport.

### Strengths, limitations and future research

A strength of this study is the longitudinal design that includes weekly measurements of 22 weeks. As such, this study gives a more reliable overview of the athletes' mental distress before, during and after PG22 compared to for example a pre-post design with only a few measurement timepoints (77). Moreover, the prospective design reduces the risk for recall and misclassification bias (66). Previous research within sports psychology has also highlighted the importance of using validated measures (9), which was done in the current study. Another strength of this study is that an accessible and user-friendly data collection method was used and that allowed for athletes with both physical and visual impairment to be included. A limitation was that no athletes with intellectual impairment competed at the Winter Paralympic Games meaning that this population should be explored in future studies. The small sample size in the present study did not allow analysis of associations and risk factors related to mental health, for example, the specific sport, impairment, performance or sports injury. It is recommended that future studies strive for lager samples by collecting data from several countries and/or over several major championships. Moreover, some athletes did not complete the questionnaire for all weeks. Future research should investigate in close collaboration with the athletes themselves how athlete monitoring could better be implemented to increase attrition (78). Another potential limitation is that only four items covering anxiety and depression were used to capture the multifaceted concept of mental distress. To obtain a more comprehensive assessment of athletes' mental distress it could be argued that more variables should be included, e.g., with questionnaires from the Sport Mental Health Assessment Tool (40). However, to avoid respondent fatigue it is important that weekly screenings are kept as short as possible (79). Furthermore, PHQ-4 has shown to have a good construct validity (80), and it could therefore be considered as a feasible tool to monitor mental distress among athletes. Finally, it should be noted that the data collection among coaches using a focus group can be a limitation if the participants have opposite opinions or controversial positions they do not want to share within a group (58). It can therefore be suggested to conduct individual interviews with coaches in further studies to get an in-depth understanding of individual variance of the research question.

## Conclusion and practical implications

Prospective data from this study revealed that elite Para athletes report similar rates of anxiety and depression as able-bodied elite athletes prior to a large competition. However, during the Paralympic Games the rate of both depression and anxiety increased, which should be considered as a concern. This demonstrate the importance of including psychological support within the high-performance team before and during large competitive events such as the Paralympic Games. To better understand the mechanisms behind, there is a need for larger epidemiological studies as well as implementation of prevention measures.

The implied practical implication from this study is that it is feasible and important to monitor Para athletes' mental distress to detect early mental distress symptoms within this population. By sharing data to coaches, athlete health monitoring can function as a “low threshold system” that enables communication of symptoms of mental distress, and especially for athletes that perceive help seeking regarding mental distress as difficult. For coaches, athlete health monitoring has the potential to reduce stressors, enhance coaching and ultimately to support athlete performance.

## Data availability statement

The datasets presented in this article are not readily available because did not collect consent to share. Requests to access the datasets should be directed to marteb@nih.no.

## Ethics statement

The studies involving human participants were reviewed and approved by Swedish Ethical Review Authority. The patients/participants provided their written informed consent to participate in this study.

## Author contributions

All authors listed have made a substantial, direct, and intellectual contribution to the work and approved it for publication.

## Conflict of interest

The authors declare that the research was conducted in the absence of any commercial or financial relationships that could be construed as a potential conflict of interest.

## Publisher's note

All claims expressed in this article are solely those of the authors and do not necessarily represent those of their affiliated organizations, or those of the publisher, the editors and the reviewers. Any product that may be evaluated in this article, or claim that may be made by its manufacturer, is not guaranteed or endorsed by the publisher.
